# Arene Ruthenium(II) Complexes Bearing the κ-*P* or κ-*P*,κ-*S* Ph_2_P(CH_2_)_3_SPh Ligand

**DOI:** 10.3390/molecules26071860

**Published:** 2021-03-25

**Authors:** Sören Arlt, Vladana Petković, Gerd Ludwig, Thomas Eichhorn, Heinrich Lang, Tobias Rüffer, Sanja Mijatović, Danijela Maksimović-Ivanić, Goran N. Kaluđerović

**Affiliations:** 1 Institute of Chemistry, Martin Luther University Halle-Wittenberg, Kurt-Mothes-Straße 2, D-06120 Halle, Germany; soeren.arlt@web.de (S.A.); ludwig.gerd7@gmail.com (G.L.); 2 Institute for Biological Research “Sinisa Stankovic” National Institute of Republic of Serbia, University of Belgrade, Bulevar despota Stefana 142, 11060 Belgrade, Serbia; vladanap@yahoo.com (V.P.); sanjamama@ibiss.bg.ac.rs (S.M.); nelamax@ibiss.bg.ac.rs (D.M.-I.); 3 Department of Engineering and Natural Sciences, University of Applied Sciences Merseburg, Eberhard-Leibnitz-Strasse 2, DE-06217 Merseburg, Germany; thomas.eichhorn@hs-merseburg.de; 4 Institute of Chemistry, Chemnitz University of Technology, Straße der Nationen 62, D-09111 Chemnitz, Germany; heinrich.lang@chemie.tu-chemnitz.de (H.L.); tobias.rueffer@chemie.tu-chemnitz.de (T.R.); 5 Faculty of Pharmacy, University of Business Academy in Novi Sad, Trg Mladenaca 5, 21000 Novi Sad, Serbia

**Keywords:** ruthenium(II), crystal structure, anticancer activity, apoptosis, autophagy

## Abstract

Neutral [Ru(η^6^-arene)Cl_2_{Ph_2_P(CH_2_)*_3_*SPh-κ*P*}] (arene = benzene, indane, 1,2,3,4-tetrahydronaphthalene: **2a**, **2c** and **2d**) and cationic [Ru(η^6^-arene)Cl(Ph_2_P(CH_2_)*_3_*SPh-κ*P*,κ*S*)]X complexes (arene = mesitylene, 1,4-dihydronaphthalene; X = Cl: **3b**, **3e**; arene = benzene, mesitylene, indane, 1,2,3,4-tetrahydronaphthalene, and 1,4-dihydronaphthalene; X = PF_6_: **4a**–**4e**) complexes were prepared and characterized by elemental analysis, IR, ^1^H, ^13^C and ^31^P NMR spectroscopy and also by single-crystal X-ray diffraction analyses. The stability of the complexes has been investigated in DMSO. Complexes have been assessed for their cytotoxic activity against 518A2, 8505C, A253, MCF-7 and SW480 cell lines. Generally, complexes exhibited activity in the lower micromolar range; moreover, they are found to be more active than cisplatin. For the most active ruthenium(II) complex, **4b**, bearing mesitylene as ligand, the mechanism of action against 8505C cisplatin resistant cell line was determined. Complex **4b** induced apoptosis accompanied by caspase activation.

## 1. Introduction

One of today’s most clinically used antitumor drug cisplatin was synthesized in 1845 by M. Peyrone. However, the structure remained unknown for the next 50 years [1,2]. A. Werner deducted the square planar structure, and the cisplatin was distinguished from the *trans* analog. Cisplatin was approved in 1978 as an antitumor agent for testicular and ovarian cancers [3,4,5]. A major disadvantage of cisplatin are its strong side effects due to its nephrotoxicity, neurotoxicity and ototoxicity [6,7]. This led to the development for alternative drugs (carboplatin, oxaliplatin, etc.) [8]. However, the side effects of the general high toxic properties of the platinum compounds were not suppressed.

At the same time, attempts were made to circumvent the side effects using nonplatinum-based cytotoxic metal compounds [9,10,11,12]. Very promising effects have been already found with titanium(IV), gallium(III), gold(III), and tin(IV) compounds [13,14,15,16,17,18,19,20].

**Figure 1:**
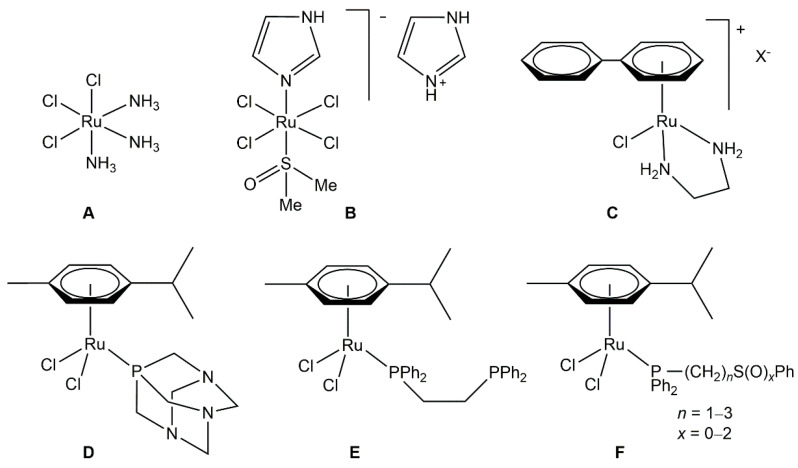
Examples of ruthenium(II/III) anticancer active compounds.

The organoruthenium(II/III) compounds seem to be particularly suitable because of their lower general toxicity in comparison with cisplatin, as well as their ability to utilize iron pathways in the body [21,22]. Keppler, Sadler and Dyson gave significant contribution in the field of ruthenium-based anticancer drugs [23,24,25]. For some ruthenium compounds, it was shown that they express a very good cytotoxic activity; importantly, particular compounds also possess an antimetastatic activity [26,27]. In some cases, they can overcome the resistance of cancer cells while the ruthenium complexes hardly affect normal cells [17]. For certain cancer lines, it has already been demonstrated that their resistance to an organic drug can be overcome by complexing to ruthenium [28]. A problem of the first anticancer active ruthenium(III)-based compound, *fac*-[Ru(NH_3_)_3_Cl_3_] (Figure 1A), is the low solubility [29]. Subsequently, water-soluble compounds such as the NAMI-A (Figure 1B) were synthesized [26]. NAMI-A shows inhibition of the formation of metastases in the lung independently of the cytostatic activity without attacking the actual tumor. Certain properties, such as faster ligand (aqua) exchange of the ruthenium in the oxidation state +2 versus +3, suggest that it is more suitable for reactions in biological systems [30,31].

It was also shown that the oxidation state +2 is stabilized through π-bonded arene ligands [32]. Existing problems such as side effects, solubility, and resistances remain in part. Several arene ruthenium(II) complexes exhibited both in vitro and in vivo promising anticancer activity. Such complexes were active in vitro in the range of 6–300 μM against human cancer cell lines (Figure 1C) [32,33]. Up to now, there are barely a few cytotoxic active ruthenium(II) complexes bearing phosphorus ligands (type D–F; Figure 1) [34,35,36]. Complex [Ru(η^6^-*p*-cymene)Cl_2_(pta)] (pta = 1,3,5-triaza-7-phosphaadamantane; Figure 1D) relived almost no anticancer activity, but on the other hand a promising antimetastatic activity [37]. Our group has explored neutral arene ruthenium(II) and iridium(III) complexes having κ*P*- and κ*P,*κ*S*-coordinated ω-diphenylphosphino-functionalized alkyl phenyl sulfide, sulfoxide, and sulfone ligands (type F, Figure 1) on their anticancer activity [34,35,38,39,40]. All complexes were found very active, importantly particular complexes showed in vitro cytotoxicities equal or higher than cisplatin.

Here, we describe the synthesis and characterization of various neutral (**2a**, **2c** and **2d**) and cationic arene ruthenium(II) complexes (**4a**–**4e**, **3b**, **3e**) with κ*P*- and κ*P,*κ*S* coordinated, respectively, 3-diphenylphosphino-functionalized propyl phenyl sulfide ligand Ph_2_P(CH_2_)*_3_*SPh. Solvolysis as well as their cytotoxic activity, especially the influence of the arene ligands, were explored. Furthermore, on the most active compound the mechanism of action against 8505C tumour cell line was elucidated.

## 2. Results and Discussion

### 2.1. Synthesis

**Scheme 1:**
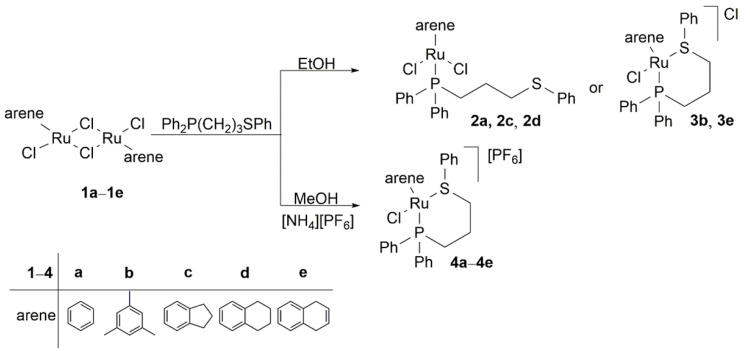
Synthesis of Ru(II) complexes.

Various binuclear ruthenium(II) complexes [{Ru(η^6^-arene)Cl_2_}_2_] (arene = benzene, mesitylene, indane, thn and 1,4-dialin) and Ph_2_P(CH_2_)_3_SPh were used for the synthesis of the **2a**–**4e** complexes as given in Scheme 1. In the reaction in which MeOH was used, a clear red solution becomes blurred over the time from which an orange precipitate of [Ru(η^6^-arene)Cl_2_{Ph_2_P(CH_2_)_3_SPh-*κ*P}] (arene = benzene, **2a**; indane, **2c**; thn (1,2,3,4-tetrahydronaphthalene), **2d**) could be collected by filtration, washed with *n*-pentane and dried in vacuum. Instead of corresponding neutral ruthenium(II) complexes **2b** (arene = mesitylene) and **2e** (arene = 1,4-dialin (1,4-dihydronaphthalene)) using the same synthetic route, cationic [Ru(η^6^-arene)Cl{Ph_2_P(CH_2_)_3_SPh-*κ*P,*κ*S}]Cl (arene = mesitylene, **3b**; 1,4-dialin, **3e**) was obtained. It was supposed that during this reaction substitution of a Cl^–^ ligand occurred with ring closure by the coordination of sulphur atom to ruthenium(II).

For the preparation of the cationic ruthenium(II) complexes of the [Ru(η^6^-arene)Cl{Ph_2_P(CH_2_)_3_SPh-*κ*P,*κ*S}][PF_6_] type, desired complexes were synthesized directly from appropriate ruthenium(II) dimers or from the corresponding neutral ruthenium(II) complexes [Ru(η^6^-arene)Cl{Ph_2_P(CH_2_)_3_SPh-*κ*P,*κ*S}]Cl (arene = benzene, **2a**; indane, **2c**; thn, **2d**) as shown in Scheme 1. Ruthenium(II) dimers, [{Ru(η^6^-arene)Cl_2_}_2_] (**1a**–**1e**), were dissolved in MeOH and allowed to react with Ph_2_P(CH_2_)_3_SPh for 3 h. Afterwards, [NH_4_][PF_6_] was added and **4a**–**4e**, [Ru(η^6^-arene)Cl{Ph_2_P(CH_2_)_3_SPh-*κ*P,*κ*S}][PF_6_] were obtained in almost quantitative yields. Using this procedure without addition of [NH_4_][PF_6_] the formation of complexes **2b** and **2e** in the reaction mixture could be proved (^31^P NMR); however, all attempts of isolation of this two complexes failed. Alternatively, neutral **2a**, **2c**, and **2d** complexes were dissolved in MeOH, and [NH_4_][PF_6_] was added yielding the appropriate cationic complexes (Scheme 1). Generally, after a short time, yellow to orange products precipitated from the reaction mixture. Neutral as well as the cationic ruthenium(II) complexes are well soluble in acetone, methanol and chloroform, but insoluble in diethyl ether and *n*-pentane.

### 2.2. Molecular Structure and Chemical Properties of the Arene Ruthenium(II) Complexes

Ruthenium(II) complexes **2a**–**4e** were characterized by microanalysis, IR and NMR (^1^H, ^13^C, ^31^P) spectroscopies and purity was determined with elemental analysis. Single-crystal X-ray structure analyses were performed for **3b**, **4d**, and **4e**.

#### 2.2.1. Crystallographic Data

**Figure 2:**
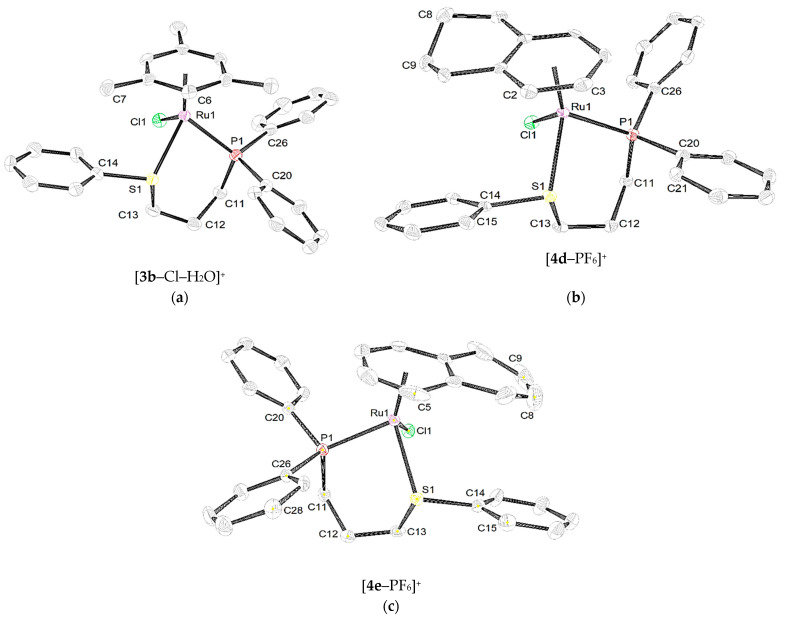
Molecular structure of [Ru(η^6^-arene)Cl{Ph_2_P(CH_2_)_3_SPh-κ*P*,κ*S*}]Cl·H_2_O (arene = mesitylene, thn and 1,4-dialin: (**a**) **3b**, (**b**) **4d**, and (**c**) **4e**, respectively). For **4e** only one independent cation is displayed. The ellipsoids are shown with a probability of 50%. H atoms have been omitted for clarity.

Single crystals of the cationic ruthenium(II) complexes [Ru(η^6^-mesitylene)Cl{Ph_2_P(CH_2_)_3_SPh-κ*P*,κ*S*}]Cl·H_2_O (**3b**) [Ru(η^6^-thn)Cl{Ph_2_P(CH_2_)_3_SPh-κ*P*,κ*S*}]PF_6_ (**4d**) and [Ru(η^6^-1,4-dialin)Cl{Ph_2_P(CH_2_)_3_SPh-κ*P*,κ*S*}]PF_6_ (**4e**) suitable for X-ray diffraction analyses were gained from methylene chloride/*n*-pentane solutions at room temperature. The compounds crystallized in discrete cations and anions. Weak C–H∙∙∙F interactions (C∙∙∙F 2.471(1)–2.823(5) Å) were found between them. Two crystallographically independent molecules were found in the unit cell of **4e**. Related bond lengths and angles differ marginally. In Figure 2 the molecular structures of the cations are shown.

Ruthenium(II) complexes are found in a half sandwich structure (“piano stool”). The coordination sphere around ruthenium(II) cation are built up by a η^6^-arene, a chloride, and κ*P*,κ*S* coordinated Ph_2_P(CH_2_)_3_SPh ligand. The angles at the ruthenium(II) atoms are close to 90° (82.4(6)–90.8(3)°). Six-membered ruthenacycles (RuPCCCS) for **3b**, **4d** and **4e** are found in chair conformation.

For all three complexes, the Ru–Cl (2.389(5)–2.412(2) Å; median Ru–Cl: 2.414 Å, lower/higher quartile: 2.389/2.442 Å, *n* = 5542), Ru–P (2.317(6)–2.342(9) Å; median Ru–P: 2.332 Å, lower/higher quartile: 2.287/2.375 Å, *n* = 2520) are in the expected range. The Ru–S bond lengths (2.367(6)–2.388(3) Å) are slightly above the usual bond lengths (median Ru–S: 2.299 Å, lower/higher quartile: 2.266/2.352 Å, *n* = 678; n—number of observations).

#### 2.2.2. Infrared Spectroscopic Data

The IR spectra of the ruthenium(II) complexes showed characteristic bands around 290 cm^–1^, which arise from Ru–Cl vibrations, while the bands found at 250 cm^–1^ are characteristic for bridging chlorido ligands in the dimers. These two bands are used for distinguishment between bridging and terminal chlorido ligands in appropriate ruthenium(II) arene complexes and can be easily used to determine the structure [41,42,43,44]. The X-ray crystallography for compounds **3b**, **4d** and **4e** confirmed assigned bands to be consistent with the assumed terminal Ru–Cl vibration. Most studies make no use of the fingerprint region and just the standard range for IR is mentioned and observed. Absorptions at around 680 cm^–1^ could be assigned to P–C vibrations of the ligand [45]. C=C- and C–H bands are found at 1400 and 1600 cm^–1^ as well as 3000 cm^–1^, respectively and are in the expected ranges for ruthenium(II) complexes [34,35]. The dominant band at 742–748 cm^–1^ derived from thioether S–C parts of the prepared complexes [46].

#### 2.2.3. NMR Data

**Figure 3:**
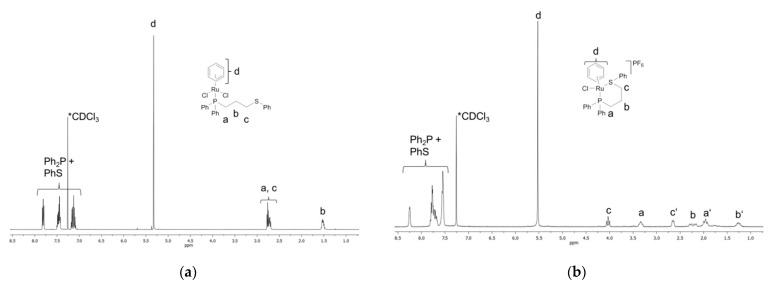
^1^H NMR spectra of Ph_2_P(CH_2_)_3_SPh, **2a** and **4a**, as an example. * solvent.

The NMR spectra confirm the constitution of the complexes and all signals were found in the expected range with correct intensities in the ^1^H NMR spectra. Thus, in the ^1^H NMR spectra (Figure 3), the resonances of the coordinating aromatic moiety (arene ligand) in the neutral complexes are found within the expected chemical shift range (5–6 ppm) but slightly upfield in comparison to the appropriate ruthenium(II) dimers. In the case of complexes bearing more complex aromatic system than benzene, the corresponding proton resonances are found at expected values. The resonances of the hydrogen atoms from the propyl chain of the Ph_2_P(CH_2_)_3_SPh ligand appeared in the range of 1 ppm to 3 ppm. The hydrogen atoms of the phenyl moieties from Ph_2_P(CH_2_)_3_SPh_2_ are resonating between 7 to 8 ppm.

As seen in Figure 2, in the ^1^H NMR spectra of the cationic complexes, additional resonances in comparison to neutral ones could be identified. In the range between 1 and 4.2 ppm, the protons of the propyl chain [Ph_2_P(CH_2_)_3_SPh] can be found. For complex **4a** the protons of the coordinated aromatic show the same resonance as for **2a**. A similar behavior was observed for the compound **4e**. The other complexes (**4b**–**4d**) showed a splitting of these resonances of the aromatic systems. The phenyl residues of the Ph_2_P(CH_2_)_3_SPh are slightly shifted in comparison to neutral complexes (7.4 to 8.2 ppm). The ^13^C NMR spectra show the same expected results as in ^1^H NMR spectra. By means of C,H-COSY NMR spectroscopy, appropriate assignment of the resonances was possible (see Figures S1–S10).

Singlets were found in the ^31^P NMR spectra of Ph_2_P(CH_2_)_3_SPh_2_ ligand. Neutral ruthenium(II) complexes (**2a**, **2c** and **2d**) [Ru(η^6^-arene)Cl{Ph_2_P(CH_2_)_3_SPh-*κ*P}] showed chemically induced shift upfield in the ^31^P NMR spectra (*ca.* 45 ppm). However, the formation of six-membered ruthenacycles (**3b**, **3e** and **4a**–**4e**) resulted in downfield shifts of up to 7.2 ppm in comparison to neutral complexes and upfield ca. 38 ppm in comparison to the phosphorous resonance in the free ligand.

In the ^31^P NMR spectra of cationic **4a**–**4e**, besides the resonances resulting from the coordinated Ph_2_P(CH_2_)_3_SPh_2_ ligand, a septet at –144 ppm was observed for the PF_6_^–^ anion.

### 2.3. Stability of Complexes in DMSO

The synthesized ruthenium(II) complexes were investigated for in vitro antitumor activity (vide infra), and hence, stability of ruthenium(II) complexes in DMSO was investigated, since DMSO was used as solubilizing agent. Earlier, Gasser et al. studied the behavior of [Ru(η^6^-arene)Cl_2_(L)] complexes (L = *N*-heterocyclic ligands) in DMSO [47].

Thus, ruthenium(II) complexes prone to dissociation in DMSO unquestionably will demonstrate changed in vitro activities in comparison to parental compounds. Recently, Keppler and co-workers showed that cyclometalated 1,2,3-triazole-derived ruthenium(II) [Ru(η^6^-arene)Cl_2_(L-κC,κN)] complexes (L = *N*-heterocyclic ligands) readily formed stable DMSO adducts in DMSO-containing solution [23]. However, using DMSO as a solubilizer in in vitro viability assay had no significant influence on the cytotoxicity.

**Figure 4:**
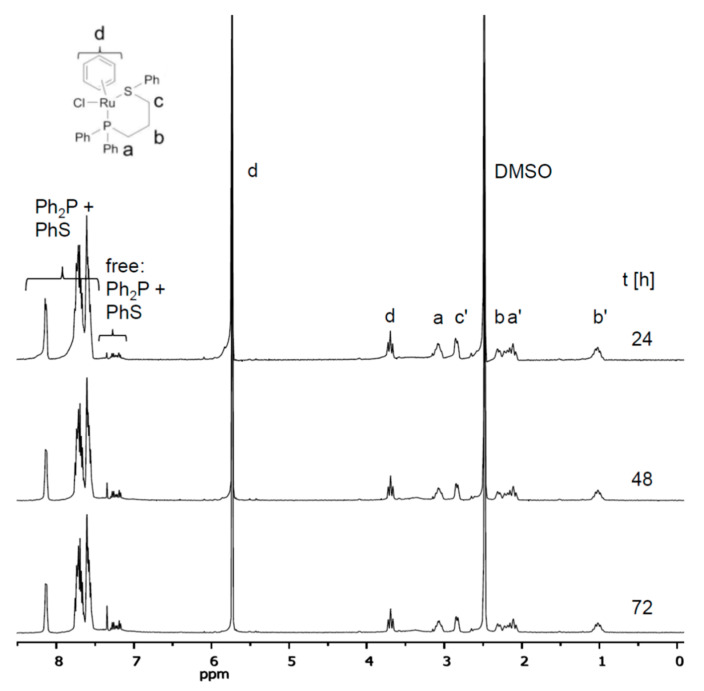
Decomposition of the **4a** cation, as an example, in DMSO-*d*_6_ observed by time dependent ^1^H NMR spectroscopy over 72 h.

All synthesized ruthenium(II) complexes are stable and storable for several weeks in air. As expected, decomposition reactions occur faster in solution than in solid state. On the basis of a solution color change (orange/red → brown/black), degradation is already visible after storage at room temperature for more than four weeks. Subsequently, such behavior is documented with ^1^H and ^31^P NMR spectroscopies. For the neutral and cationic ruthenium(II) complexes, a similar behavior in DMSO was observed. The investigation **4a** stability in DMSO, as an example, over 72 h is presented in Figure 4. The ^1^H NMR spectra over time clearly indicate that **4a** degrades to low extend for investigated period of time. Thus, for the in vitro studies **4a** is acting on the cells.

Within 72 h, there were appearances of new chemical shifts with low intensity detectable in the aromatic region, ascribed to free Ph_2_P(CH_2_)_3_SPh, in both ^1^H and ^31^P NMR spectra. However, after 72 h for neutral and cationic ruthenium(II) complexes, much clearer appearance of degradation products could be identified. Apart from the chemical shifts belonging to the free Ph_2_P(CH_2_)_3_SPh ligand observed in the ^1^H NMR, consequently, the new resonances are also noted in the ^31^P NMR spectra. The decomposition reactions also occur in different solvents. For instance, degradation in chloroform (it might be due to chlorination, often via a radical pathway) is much faster than in DMSO. Moreover, significant decompositions in chloroform could be observed, for example, after only 12 h for the complex **4a**. To summarize, the decomposition of ruthenium(II) complexes in DMSO occurs only after days.

### 2.4. Cytotoxicity Study

**Figure 5:**
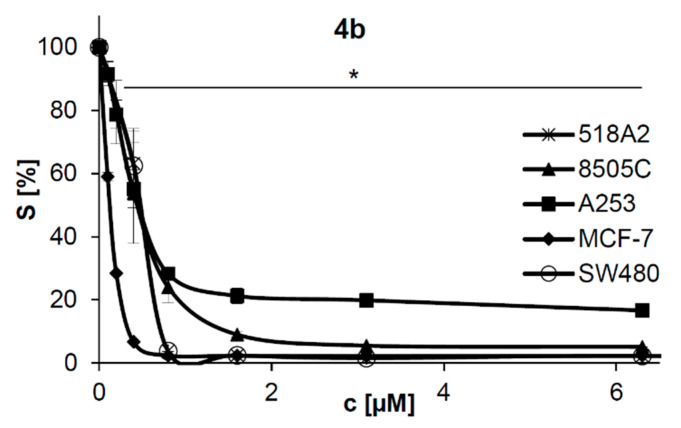
Representative graphs showing the survival (in %) of cells growth for 96 h in the presence of increasing concentrations of **4b** (as an example, * *p* < 0.05).

To evaluate the efficacy of the new neutral (**2a**, **2c**, **2d**) and cationic ruthenium(II) complexes (**4a**–**4e**) human 518A2 (melanoma), 8505C (anaplastic thyroid tumor), A253 (head and neck tumor), MCF-7 (breast), and SW480 (colon) cell lines were treated with diverse concentrations of ruthenium(II) complexes for 96 h. The viabilities of cells were assessed using sulforhodamine-B (SRB) microculture colorimetric assay [48]. All ruthenium(II) complexes exhibited a dose-dependent inhibition of the cell growth (Figure 5). The IC_50_ values are summarized in Table 1. Additionally, for comparison, the respective activities of analogous complexes having *p*-cymene as arene ligand [Ru(η^6^-*p*-cymene)Cl_2_{Ph_2_P(CH_2_)_3_SPh-κ*P*}] (**2f**) and [Ru(η^6^-*p*-cymene)Cl{Ph_2_P(CH_2_)_3_SPh-κ*P*,κ*S*}][PF_6_] (**4f**) as well as cisplatin are included.

As shown in our previous study, the ligand Ph_2_P(CH_2_)_3_SPh showed much lower antitumor activity (IC_50_ = 10.7–26.8 µM) than the investigated ruthenium(II) complexes. κ*P* or κ*P*,κ*S* coordination of Ph_2_P(CH_2_)_3_SPh to [Ru(η^6^-arene)Cl_2_] or [Ru(η^6^-arene)Cl] moiety, respectively, makes the obtained ruthenium(II) complexes extremely active against all tumor cell lines. Most of the complexes exhibited much higher activity than cisplatin, up to 17 times. Thus, the most active cationic ruthenium(II) complex bearing mesitylene ligand (**4b**) showed the highest cytotoxic potency against cisplatin resistant MCF-7 cell line. For few ruthenium(II) complexes, similar cytotoxic effects were found as the reference compound cisplatin, and only one was less active (neutral complex **2c** against 518A2). From the neutral complexes, the most active was found to be **2a**, while from the cationic, **4b**. Benzene, indane and thn aryl ligands bound to a [RuCl_2_{Ph_2_P(CH_2_)_3_SPh-κ*P*}] or [RuCl{Ph_2_P(CH_2_)_3_SPh-κ*P*,κ*S*}]^+^ moiety exhibit similar effects on the in vitro anticancer activity. Complexes **2a**, **2c**, and **2d** in comparison to the corresponding neutral ruthenium(II) complex bearing the *p*-cymene ligand showed superior activities up to 4.1/3.4 times against 8505C/MCF-7 and up to 6.7 times against cisplatin sensitive A253 [34,35]. Cationic complexes **4a**, **4c**, and **4d** exhibited mainly higher activity than the appropriate ruthenium(II) complex having the *p*-cymene ligand on 518A2 and 8505C cell lines.

**Figure 6:**
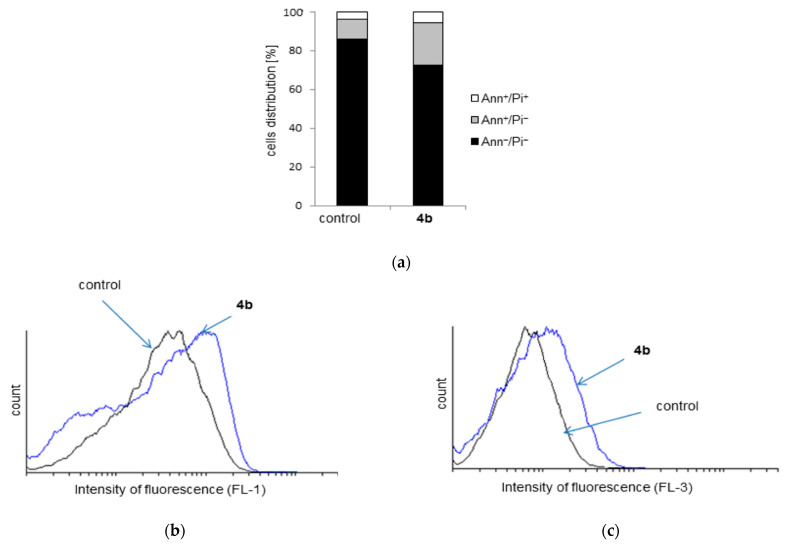
8505C cells treated with **4b**: (**a**) apoptosis, (**b**) caspase activation, and (**c**) induction of autophagy after 72 h of treatment with IC_50_ dose.

For further analysis, 8505C anaplastic thyroid carcinoma, resistant to chemotherapy, was selected. To define the cause of decreased number of viable cells in cultures exposed to IC_50_ dose of **4b**, presence of apoptotic as well as necrotic cells was estimated by Annexin V-FITC/PI staining. As could be seen in Figure 6a, cultivation in the presence of **4b** elevated percentage of early apoptotic cells, marked as Ann^+^/PI^−^. In addition, occurrence of late apoptotic, double positive cells (Ann^+^/PI^+^), was found in cultures exposed to **4b**.

These cells are rather secondary than primary necrotic, having in mind that apoptotic cells in culture must necrotize at the end point. Obtained results indicated that **4b** induced apoptosis of 8505C. Subsequently, apostat staining showed that apoptosis triggered by the investigated drug in 8505C cells was accompanied by caspase activation (Figure 6b). While autophagy often follows the apoptosis as a cell attempt to repair damage, but also, under some circumstances, the same process could mediate cell removal herein. The amount of autophagosomes in cytoplasm of 8505C cells treated with **4b** was quantified using supravital dye acridine orange (AO). Flow-cytometric analysis (Figure 6c) revealed slightly elevated fluorescence upon the treatment with **4b.**, thus pointing out the irrelevance of autophagic process to the drugs antitumor action. The same mode of action was found for complexes **2f** and **4f** [40]; however, the compound described herein is superior to those already published since an IC_50_ dose of **4b** causes similar effect as double IC_50_ doses of complexes **2f** and **4f**.

## 3. Materials and Methods

### 3.1. General Manipulations

Reactions were performed under argon using standard Schlenk techniques. Solvents were dried (methylene chloride over CaH_2_; diethyl ether/*n*-pentane over Na/benzophenone; ethanol over magnesiumethanolate) and freshly distilled prior to use. NMR spectra (^1^H, ^13^C, ^31^P) were recorded at 27 °C with Varian Gemini 2000 (400 MHz) and Inova 500 (500 MHz) spectrometers. Chemical shifts are relative to solvent signals (CDCl_3_, δ_H_ 7.26, δ_C_ 77.0) as internal references; δ(^31^P) is relative to external H_3_PO_4_ (85%). Microanalyses (C, H, S) were performed in the Microanalytical Laboratory of the University of Halle using a CHNS-932 (LECO) elemental analyzer. IR spectra were recorded with Bruker Tensor 27 FT-IR Spectrometer from 4000–250 cm^–1^. [{RuCl_2_(η^6^-arene)}_2_] (arene = benzene, mesitylene, indane, thn (1,2,3,4-tetrahydronaphthalene), and 1,4-dialin (1,4-dihydronaphthalene): **1a**–**1e**, respectively) and the ligand Ph_2_P(CH_2_)*_3_*SPh were prepared according to literature procedures [34,43].

### 3.2. Synthetic Procedures

#### 3.2.1. Preparation of [Ru(η^6^-arene)Cl_2_{Ph_2_P(CH_2_)_3_SPh-κP}] (**2a**, **2c**, **2d**) and [Ru(η^6^-arene)Cl{Ph_2_P(CH_2_)_3_SPh-κP,κS}]Cl (**3b**, **3e**)

To an ethanol solution (50 mL) of respective dimer [{RuCl_2_(η^6^-arene)}_2_] (1a–1e; 50 mg) Ph_2_P(CH_2_)*_3_*SPh (2.2 equiv.) was added while stirring for 3 (**2a**, **2c** or **2d**) or 5 h (**3b**, **3e**). Afterwards, the obtained precipitate was filtered off, washed with *n*-pentane (2 × 5 mL), and dried in vacuum.

**2a**. Yield: 85 mg (73%). Anal. Found: C, 55.03; H, 4.47; S, 5.72. Calcd for C_31_H_35_Cl_2_PRuS (586.52): C, 55.29; H, 4.64; S, 5.47. ^1^H NMR (500 MHz, CDCl_3_): δ 7.85–7.77 (m, 4H, C*H*_Ph_), 7.52–7.40 (m, 6H, C*H*_Ph_), 7.20–7.06 (m, 5H, C*H*_Ph_), 5.33 (s, 6H, C*H*), 2.83–2.62 (m, 4H, C*H*_2_PPh_2_; C*H*_2_SPh), 1.57–1.46 (m, 2H, CH_2_C*H*_2_CH_2_). ^13^C NMR (126 MHz, CDCl_3_): δ 136.0–125.7 (18 × *C*_Ph_), 88.5 (6 × *C*H), 34.3 (*C*H_2_SPh), 23.3 (*C*H_2_PPh_2_), 23.0 (CH_2_*C*H_2_CH_2_). ^31^P NMR (202 MHz, CDCl_3_): δ 24.5 (^1^*J*_P,CH2_ = 29.6 Hz). IR (υ, cm^–1^): 291(s), 349(m), 428(m), 453(m), 497(s), 519(m), 650(m), 692(m), 742(s), 810(m), 850(w), 987(m), 1097(m), 1169(w), 1435(m), 1475(w), 1624(w), 2912(w), 2991(w), 3053(w).

**2c**. Yield: 67 mg (62%). Anal. Found: C, 57.21; H, 4.72; S, 5.41. Calcd for C_30_H_31_Cl_2_PRuS (626.58): C, 57.51; H, 4.99; S, 5.12. ^1^H NMR (500 MHz, CDCl_3_): δ 7.84–7.67 (m, 4H, C*H*_Ph_), 7.49–7.37 (m, 6H, C*H*_Ph_), 7.20–7.06 (m, 5H, C*H*_Ph_), 5.12–5.06 (m, 2H, C*H*), 4.56–4.50 (m, 2H, C*H*), 3.02–2.95 (m, 2H, C*H*_2_PPh_2_), 2.82–2.70 (m, 4H, C*H*_2_SPh; C*H*_2_), 2.64–2.53 (m, 2H, C*H*_2_), 2.34–2.22 (m, 1H, C*H*_2_), 2.07–1.98 (m, 1H, C*H*_2_), 1.62–1.47 (m, 2H, CH_2_C*H*_2_CH_2_). ^13^C NMR (126 MHz, CDCl_3_): δ 133.0–128.4 (18 × *C*_Ph_), 111.2 (2 × *C*_i_), 83.4 (2 × *C*H), 82.6 (2 × *C*H)H, 34.2 (*C*H_2_SPh), 29.7 (2 × *C*H_2_), 23.9 (*C*H_2_PPh_2_), 23.1 (*C*H_2_), 22.7 (CH_2_*C*H_2_CH_2_). ^31^P NMR (202 MHz, CDCl_3_): δ 25.5 (^1^*J*_P,CH2_ = 30.9 Hz, ^1^*J*_P,C_ = 9.6 Hz). IR (υ, cm^–1^): 285(s), 331(m), 349(m), 380(m), 435(s), 453(m), 495(s), 522(m), 581(w), 617(w), 650(m), 692(s), 712(m), 746(s), 810(m), 875(m), 910(w), 987(m), 1043(w), 1066(w), 1095(m), 1193(w), 1250(w), 1311(w), 1421(m), 1481(w), 1581(w), 1600(w), 2862(w), 2920(w), 2976(w), 3049(w).

**2d**·**H_2_O**. Yield: 79 mg (75%). Anal. Found: C, 57.93; H, 4.85; S, 5.37. Calcd for C_31_H_35_Cl_2_OPRuS (640.61): C, 58.12; H, 5.19; S, 5.00. ^1^H NMR (400 MHz, CDCl_3_): δ 7.74–7.56 (m, 4H), 7.53–7.35 (m, 6H), 7.29–7.05 (m, 5H), 5.78–5.68 (m, 2H), 5.57–5.46 (m, 2H), 2.92–2.53 (m, 6H, C*H*_2_PPh_2_; C*H*_2_SPh; C*H*_2_), 2.45–2.22 (m, 2H, C*H*_2_), 1.83–1.58 (m, 4H, C*H*_2_; CH_2_C*H*_2_CH_2_), 1.48–1.33 (m, 2H, C*H*_2_). ^13^C NMR (101 MHz, CDCl_3_): δ 132.9–128.3 (18 × *C*_Ph_), 94.6 (2 × *C*_i_), 82.0 (2 × *C*H), 80.9 (2 × *C*HHH), 34.4 (*C*H_2_SPh), 26.3 (2 × *C*H_2_), 26.0 (*C*H_2_PPh_2_), 21.3 (CH_2_*C*H_2_CH_2_), 21.1 (2 × *C*H_2_). ^31^P NMR (162 MHz, CDCl_3_): δ 28.4 (^1^*J*_P,CH2_ = 30.5 Hz). IR (υ, cm^–1^): 298(s), 380(m), 436(m), 457(m), 490(m), 532(m), 580(w), 632(w), 700(m), 725(m), 748(s), 798(w), 815(w), 852(m), 905(w), 982(w), 1093(m), 1155(w), 1196(w), 1298(w), 1414(m), 1437(m), 1479(w), 1516(w), 1578(w), 2866(w), 2933(w), 3057(w).

**3b**·**H_2_O**. Yield: 66 mg (60%). Anal. Found: C, 55.68; H, 5.22; S, 4.83. Calcd for C_30_H_35_Cl_2_OPRuS (646.61): C, 55.72; H, 5.46; S, 4.95. ^1^H NMR (500 MHz, CDCl_3_): δ 8.52–7.37 (m, 15H, C*H*_Ph_), 5.23 (s, 3H, C*H*), 4.05 (t, ^2^*J*_H,H_ = 12.0 Hz, 1H, C*H*_2_SPh), 3.31 (m, 1H, C*H*_2_PPh_2_), 2.60 (d, ^2^*J*_H,H_ = 12.0 Hz, 1H, C*H*_2_SPh), 2.30–1.90 (m, 2H, C*H*_2_PPh_2_; CH_2_C*H*_2_CH_2_), 1.80 (s, 9H), 1.28–1.13 (m, 1H, CH_2_C*H*_2_CH_2_). ^13^C NMR (126 MHz, CDCl_3_): δ 135.5–128.7 (18 × *C*_Ph_), 110.4 (3 × *C*_i_), 89.3 (3 × *C*H), 36.0 (*C*H_2_SPh), 25.2 (*C*H_2_PPh_2_), 21.9 (CH_2_*C*H_2_CH_2_), 18.8 (3x*C*H_3_). ^31^P NMR (202 MHz, CDCl_3_): δ 22.1 (^1^*J*_P,CH2_ = 31.1 Hz, ^1^*J*_P,C_ = 10.5 Hz). IR (υ, cm^–1^): 299(m), 349(w), 434(m), 453(m), 488(m), 501(m), 521(m), 642(m), 692(m), 744(m), 808(m), 860(w), 989(m), 1039(m), 1097(m), 1169(w), 1298(w), 1381(w), 1437(m), 1477(w), 1539(w), 2910(w), 2966(w), 3051(w).

**3e**·**H_2_O**. Yield: 80 mg (74%). Anal. Found: C, 56.48; H, 4.72; S, 5.02. Calcd for C_31_H_33_Cl_2_OPRuS (656.61): C, 56.71; H, 4.76; S, 4.88. ^1^H NMR (500 MHz, CDCl_3_): δ 8.48–7.37 (m, 15H, C*H*_Ph_), 6.51 (s (br), 1H, C*H*), 5.80 (s (br), 1H, C*H*), 5.56–5.32 (m, 3H, C*H*), 4.56 (s, 1H, C*H*) 4.12 (t, ^2^*J*_H,H_ = 11.9 Hz, 1H, C*H*_2_SPh), 3.38 (s (br), 1H, C*H*_2_PPh_2_), 2.56 (‘ddd’, 5H, C*H*_2_PPh_2_; C*H*_2_SPh; C*H*_2_), 2.26–2.10 (m, 1H, C*H*_2_), 2.06–1.93 (m, 1H, CH_2_C*H*_2_CH_2_), 1.24 (s (br), 1H, C*H*_2_). ^13^C NMR (126 MHz, CDCl_3_): δ 132.4–128.9 (18 × *C*_Ph_), 122.5 (*C*H), 121.8 (*C*H), 105.6 (2 × *C*_i_) 89.3 (2 × *C*H), 88.2 (2 × *C*H), 33.8 (*C*H_2_SPh), 26.4 (2 × *C*H_2_), 26.2 (*C*H_2_PPh_2_), 21.4 (CH_2_*C*H_2_CH_2_). ^31^P NMR (202 MHz, CDCl_3_): δ 22.2 (s). IR (υ, cm^–1^): 295(m), 351(w), 449(m), 487(m), 525(m), 645(m), 688(m), 743(s), 820(m), 854(w), 989 (w), 1096(m), 1169(w), 1273(w), 1390(w), 1437(m), 1480(w), 1525(w), 1660(w), 2925(w), 2960(w), 3053(w).

#### 3.2.2. Preparation of [Ru(η^6^-arene)Cl{Ph_2_P(CH_2_)_3_SPh-κP,κS}][PF_6_] (**4a**–**4e**)

To a methanol solution (50 mL) of respective [{RuCl_2_(η^6^-arene)}_2_] (50 mg) Ph_2_P(CH_2_)*_3_*SPh (2.2 mmol) was added while stirring. As shown by ^31^P NMR spectroscopy, complexes **2b** and **2e** were formed [δ: **2b**, 29.1 (s); **2e**, 27.8 (s)], however, isolation failed. After 3 h, [NH_4_][PF_6_] (6 equiv.) was added and the reaction mixture was stirred for additional 3 h. The obtained precipitate was filtered off, washed with diethyl ether (2 × 5 mL) and dried in vacuum.

Alternatively, to a an anhydrous methanol solution (50 mL) of the appropriate neutral complex (**2a**, **2c**, or **2d**) [NH_4_][PF_6_] (6 equiv.) was added and the reaction solution was stirred at room temperature for 3 h. Then, the obtained precipitate was filtered off, washed with *n*-pentane (2 × 5 mL) and dried in vacuum.

**4a**. Yield: 58 mg (99%). Anal. Found: C, 46.48; H, 3.98. Calcd for C_27_H_27_ClF_6_P_2_RuS (696.03): C, 46.59; H, 3.91. ^1^H NMR (400 MHz, CDCl_3_): δ 8.32–7.47 (m, 15H, C*H*_Ph_), 5.53 (s, 6H, C*H*), 4.03 (t,^2^*J*_H,H_ = 11.7 Hz, 1H, C*H*_2_SPh), 3.40–3.26 (m, 1H, C*H*_2_PPh), 2.73–2.61 (m, 1H, C*H*_2_SPh), 2.33–2.13 (m, 1H, C*H*_2_PPh), 2.03–1.86 (m, 1H, CH_2_C*H*_2_CH_2_), 1.33–1.18 (m, 1H, CH_2_C*H*_2_CH_2_). ^13^C NMR (126 MHz, CDCl_3_): δ 137.7–129.1 (18 × *C*_Ph_), 92.9 (*C*H), 33.3 (*C*H_2_SPh), 26.9 (*C*H_2_PPh), 21.3 (CH_2_*C*H_2_CH_2_). ^31^P NMR (202 MHz, CDCl_3_): δ 20.5 (^1^*J*_P,CH2_ = 32.6 Hz, ^1^*J*_P,C_ = 10.4 Hz), –144.1 (sep, ^1^*J*_P,F_ = 713 Hz, *P*F_6_). IR (υ, cm^–1^): 293(m), 314(m), 356(m), 455(m), 484(s), 495(m), 520(m), 555(s), 648(m), 690(m), 744(s), 829(s), 900(w), 987(w), 1026(w), 1097(w), 1169(w), 1261(w), 1313(w), 1392(w), 1438(m), 1481(w), 1579(w), 2864(w), 2916(w), 3057(w).

**4b**. Yield: 56 mg (98%). Anal. Found: C, 49.17; H, 4.48. Calcd for C_30_H_33_ClF_6_P_2_RuS (738.11): C, 48.82; H, 4.51. ^1^H NMR (400 MHz, CDCl_3_): δ 8.36–7.37 (m, 15H, C*H*_Ph_), 4.96 (s, 3H), 4.04 (t, ^2^*J_H,H_* = 12.3 Hz, 1H, C*H*_2_SPh), 3.30 (s (br), 1H, C*H*_2_PPh), 2.70–2.53 (m, 1H, C*H*_2_SPh), 2.28–1.93 (m, 2H, C*H*_2_PPh; CH_2_C*H*_2_CH_2_), 1.75 (s, 9H, C*H*_3_), 1.33–1.12 (m, 1H, CH_2_C*H*_2_CH_2_). ^13^C NMR (101 MHz, CDCl_3_): δ 135.1–128.7 (18 × *C*_Ph_), 110.4 (*C*_i_), 89.01 (*C*H), 36.03 (*C*H_2_SPh), 25.53 (*C*H_2_PPh), 21.84 (CH_2_*C*H_2_CH_2_), 18.38 (*C*H_3_). ^31^P NMR (202 MHz, CDCl_3_): δ 21.9 (^1^*J*_P,C_ = 10.1 Hz), –144.0 (sep, ^1^*J*_P,F_ = 712 Hz, *P*F_6_). IR (υ, cm^–1^): 300(m), 349(w), 434(m), 451(m), 486(s), 522(m), 555(s), 615(w), 644(m), 690(m), 744(m), 831(s), 877(w), 916(w), 989(w), 1034(w), 1097(w), 1168(w), 1268(w), 1300(w), 1394(w), 1439(m), 1479(w), 1537(w), 1578(w), 2912(w), 2976(w), 3060(w).

**4c**. Yield: 57 mg (97%). Anal. Found: 49.13; H, 4.21. Calcd for C_30_H_33_ClF_6_P_2_RuS (736.09): C, 48.95; H, 4.24. ^1^H NMR (500 MHz, CDCl_3_): δ 8.41–7.40 (m, 15H, C*H*_Ph_), 5.80 (t, ^3^*J*_H,H_ = 5.7 Hz, 1H, C*H*), 5.60 (m, 1H, C*H*), 5.49 (d, ^3^*J*_H,H_ = 5.7 Hz, 1H, C*H*), 4.80 (‘dd’, 1H, C*H*), 4.10 (t, ^2^*J*_H,H_ = 11.8 Hz, 1H, C*H*_2_SPh), 3.44–3.31 (m, 1H, C*H*_2_PPh), 2.67 (‘dd’, 1H, C*H*_2_SPh), 2.472.31 (m, 1H, C*H*_2_PPh), 2.32–2.12 (m, 2H, C*H*_2_), 2.06–1.89 (m, 2H, CH_2_C*H*_2_CH_2_; C*H*_2_), 1.82–1.70 (m, 2H, C*H*_2_), 1.68 –1.59 (m, 1H, C*H*_2_), 1.31–1.10 (m, 1H, CH_2_C*H*_2_CH_2_). ^13^C NMR (126 MHz, CDCl_3_): δ 135.1–128.8 (18 × *C*_Ph_), 92.5 (*C*_i_), 88.0 (*C*H), 84.6 (*C*H), 33.4 (*C*H_2_SPh), 29.7 (*C*H_2_), 29.3 (*C*H_2_), 24.1 (*C*H_2_PPh), 22.6 (*C*H_2_), 21.3 (CH_2_*C*H_2_CH_2_).^31^P NMR (202 MHz, CDCl_3_): δ 20.6 (^1^*J*_P,CH2_ = 30.7 Hz, ^1^*J*_P,C_ = 10.5 Hz), –144.1 (sep, ^1^*J*_P,F_ = 712 Hz, *P*F_6_). IR (υ, cm^–1^): 285(s), 326(m), 349(w), 376(w), 438(m), 482(m), 523(m), 555(s), 646(m), 692(s), 710(m), 746(s), 829(s), 872(m), 910(w), 991(w), 1097(w), 1169(w), 1259(w), 1396(w), 1419(w), 1441(m), 1472(w), 1574(w), 2910(w), 2972(w), 3043(w).

**4d**. Yield: 55 mg (94%). Anal. Found: C, 49.63; H, 4.40. Calcd for C_31_H_33_ClF_6_P_2_RuS (750.12): C, 49.63; H, 4.43. ^1^H NMR (400 MHz, CDCl_3_): δ 8.28–7.42 (m, 15H, C*H*_Ph_), 5.80 (t, ^3^*J*_H,H_ = 5.5 Hz, 1H, C*H*), 5.50 (dd, *J* = 9.4, 5.2 Hz, 1H, C*H*), 5.28 (d, ^3^*J*_H,H_ = 5.7 Hz, 1H, C*H*), 4.52–4.45 (m, 1H, C*H*), 4.12 (t, ^2^*J*_H,H_ = 11.8 Hz, 1H, C*H*_2_SPh), 3.45–3.32 (m, 1H, C*H*_2_PPh), 2.73–2.62 (m, 1H, C*H*_2_SPh), 2.31–2.09 (m, 2H, C*H*_2_), 2.03–1.90 (m, 2H, CH_2_C*H*_2_CH_2_; C*H*_2_), 1.84–1.56 (m, 3H, C*H*_2_), 1.47–1.35 (m, 2H, C*H*_2_), 1.32–1.14 (m, 1H, CH_2_C*H*_2_CH_2_). ^13^C NMR (101 MHz, CDCl_3_): δ 135.1–128.9 (18 × *C*_Ph_), 96.0 (*C*_i_), 88.5 (*C*H), 86.0 (*C*H), 85.4 (*C*H), 33.6 (*C*H_2_SPh), 25.8 (*C*H_2_), 25.7 (*C*H_2_), 24.6 (*C*H_2_PPh), 21.3 (CH_2_*C*H_2_CH_2_), 20.5 (*C*H_2_). ^31^P NMR (162 MHz, CDCl_3_): δ 21.5 (^1^*J*_P,CH2_ = 30.3 Hz, ^1^*J*_P,C_ = 10.5 Hz), –144.1 (sep, ^1^*J*_P,F_ = 710 Hz, *P*F_6_). IR (υ, cm^–1^): 283(m), 347(m), 438(m), 486(m), 523(m), 555(s), 646(m), 692(m), 744(m), 756(m), 829(s), 904(w), 989(w), 1097(w), 1167(w), 1261(w), 1398(w), 1439(m), 1574(w), 2864(w), 2912(w), 2949(w), 3055(w).

**4e**. Yield: 54 mg (95%). Anal. Found: C, 50.00; H, 4.17. Calcd for C_31_H_31_ClF_6_P_2_RuS (748.10): C, 49.77; H, 4.18. ^1^H NMR (500 MHz, CDCl_3_): δ 8.26–7.41 (m, 15H, C*H*_Ph_), 5.84 (s, 1H, C*H*), 5.52–5.29 (m, 4H, C*H*), 4.70–4.57 (m, 1H, C*H*), 4.10 (t, ^2^*J*_H,H_ = 12.1 Hz, 1H, C*H*_2_SPh), 3.39 (s (br), 1H, C*H*_2_PPh), 2.83 (d, *J*_H,H_ = 22.2 Hz, 1H, C*H*_2_), 2.75–2.62 (m, 2H, C*H*_2_SPh; C*H*_2_), 2.49 (d, *J*_H,H_ = 22.2 Hz, 1H, C*H*_2_), 2.34–2.12 (m, 2H, C*H*_2_PPh; C*H*_2_), 2.06–1.92 (m, 1H, CH_2_C*H*_2_CH_2_), 1.31–1.14 (m, 1H, CH_2_C*H*_2_CH_2_). ^13^C NMR (126 MHz, CDCl_3_): δ 135.2–129.0 (18 × *C*_Ph_), 122.2 (*C*H), 121.8 (*C*H), 92.89(*C*_i_), 89.5 (*C*H), 87.8 (*C*H), 85.1 (*C*H), 34.3 (*C*H_2_SPh), 26.1 (*C*H_2_), 24.3 (*C*H_2_PPh), 21.4 (CH_2_*C*H_2_CH_2_). ^31^P NMR (162 MHz, CDCl_3_): δ 22.0 (^1^*J*_P,CH2_ = 30.8 Hz, ^1^*J*_P,C_ = 10.6 Hz), –144.1 (sep, ^1^*J*_P,F_ = 712 Hz, *P*F_6_). IR (υ, cm^–1^): 291(m), 353(w), 449(m), 484(m), 523(m), 555(s), 656(m), 690(s), 742(s), 827(s), 989(w), 1097(w), 1169(w), 1263(w), 1315(w), 1398(w), 1438(m), 1481(w), 1523(w), 1578(w), 1670(w), 2918(w), 3059(w).

### 3.3. Crystallography

Data for X-ray diffraction analyses of single crystals of **3b**·H_2_O, **4d**, and **4e** were collected on an Rigaku Oxford Gemini S diffractometer at 110K using Mo-*K*α radiation (λ = 0.71073 Å, graphite monochromator, CrysAlis Pro Version 1.171.36.28). Absorption corrections were applied multiscanning with the SCALE3 ABSPACK algorithm (*T*_min_/*T*_max_: 0.89/1.00, **3b**·H_2_O; 0.84/1.00 **4d**; 0.98/1.00, **4e**), respectively, of the CrysAlisPro software package. The structures were solved with direct methods using SHELXS-2013 and refined using full-matrix least-square routines against *F*^2^ with SHELXL-2013 [49]. All non-hydrogen atoms were refined with anisotropic displacement parameters and hydrogen atoms with isotropic ones. Carbon-bonded hydrogen atoms were placed in calculated positions according to the riding model. The hydrogen atom positions of the water molecule of **3b**·H2O were taken from difference Fourier maps and refined with DFIX and DANG constraints. CCDC 1907326-1907328 contains the supplementary crystallographic data for this paper. These data can be obtained free of charge from the Cambridge Crystallographic Data Centre via www.ccdc.cam.ac.uk/data_request/cif (accessed on 29 December 2020).

### 3.4. In Vitro Studies

#### 3.4.1. Reagents and Cells

Fetal calf serum (FCS), RPMI-1640, phosphate-buffered saline (PBS) and dimethyl sulfoxide (DMSO) were obtained from Sigma (St. Louis, MO, USA). Acridin orange (AO) was from Labo-Moderna (Paris, France). Annexin V-FITC (AnnV) and apostat were purchased from Biotium (Hayward, CA, USA) and R&D (R&D Systems, Minneapolis, MN, USA), while penicillin/streptomycin from PAA Laboratories.

The cell lines 518A2, 8505C, A253, MCF-7 and SW480 were routinely maintained as monolayers in nutrient medium (RPMI-1640 supplemented with 10% FCS, 2 mM l-glutamine, 0.01% sodium pyruvate and 1% penicillin/streptomycin) at 37 °C in a humidified atmosphere with 5% CO_2_. Stock solutions of investigated compounds were prepared in DMSO at a concentration of 20 mM, filtered through Millipore filter, 0.22 μm, before use, and diluted by nutrient medium to various working concentrations. After standard trypsinization, cells were seeded at 2.5 × 10^3^ cells/well in 96-well plates for viability determination and 1.5 × 10^5^ cells/well in 6-well plate for flow cytometry.

#### 3.4.2. Determination of Cell Viability by Sulphorhodamine Assay (SRB)

The viability of adherent viable cells was measured by SRB assay [48]. Cells were exposed to a wide range of doses of the drugs for 96 h and then fixed with 10% of TCA for 2 h at 4 °C. After fixation, cells were washed in distilled water, stained with 0.4% SRB solution 30 min at RT, washed, and dried overnight. The dye was dissolved in 10 mM TRIS buffer, and the absorbance was measured at 540 nm with the reference wavelength at 640 nm. IC50 values, defined as the concentrations of the compound at which 50% of cell inhibition occurs ± SD were calculated using four-parameter logistic function and presented as mean from three independent experiments.

#### 3.4.3. AnnexinV-FITC/PI, AO Staining and Caspase Detection

Cells were exposed to IC_50_ dose of **4b** for 72 h. After trypsinization cells were stained with AnnV-FITC/PI (Biotium, Hayward, CA, USA) or apostat according to the manufacturer’s instruction. Alternatively, cells were stained with solution of 100 μM AO 15 min at 37 °C. Cells were analyzed with CyFlow^®^ Space Partec with Partec FloMax^®^ software.

## 4. Conclusions

In this work, the synthesis of various neutral and cationic ruthenium complexes of the general formulae [Ru(η^6^-arene)Cl_2_{Ph_2_P(CH_2_)_3_SPh-*κ*P}] and [Ru(η^6^-arene)Cl{Ph_2_P(CH_2_)_3_SPh-*κ*P,*κ*S}]X (arene **=** benzene, mesitylene, indane, thn, and 1,4-dialin; X = Cl^−^ or PF_6_^−^), respectively, was established. Complexes were characterized by IR and multinuclear NMR spectroscopy. Moreover, crystal structures of **3b**, **4d**, and **4e** complexes were obtained and confirmed proposed structures. The stability of the complexes in DMSO, thus possibility of DMSO to replace ligands, was investigated using NMR spectroscopy. Solvolysis is considerably hindered within the first 3 days, therefore the applied ruthenium(II) complexes in in vitro investigations did not suffer with the DMSO substitution in the stock solution.

The cytotoxicity of all arene ruthenium(II) complexes was determined in five human cancer cell lines (518A2, 8505C, A253, MCF-7 and SW480). All ruthenium(II) complexes demonstrated high cytotoxic potential with the IC_50_ values down to the low micromolar range. Selected cationic ruthenium(II) complex bearing the mesytil moiety (**4b**) was found to induce apoptosis in 8505C cisplatin resistant cell line. This process was associated with caspase activation. Taken together, herein are synthesized ruthenium(II) complexes with strong anticancer potential, whose mechanism of action is based on the caspase triggered apoptosis, thus encouraging future development of this promising ruthenium(II) complexes.

## Figures and Tables

**Table 1:** IC_50_ [µM] values of complexes **2a**, **2c**, **2d**, **4a**–**4e** as well as **2f** [34] and **4f** [35] and cisplatin.

**Compound**	**518A2**	**8505C**	**A253**	**MCF-7**	**SW480**
**2a**	0.77 ± 0.04	0.88 ± 0.05	0.64 ± 0.05	0.52 ± 0.06	1.23 ± 0.08
**2c**	2.16 ± 0.04	1.03 ± 0.08	0.59 ± 0.06	0.70 ± 0.04	1.91 ± 0.15
**2d**	0.81 ± 0.04	2.98 ± 0.15	0.98 ± 0.12	0.78 ± 0.06	1.56 ± 0.10
**2f**	3.02 ± 0.06	3.64 ± 0.13	3.94 ± 0.11	1.75 ± 0.45	2.68 ± 0.10
**4a**	1.35 ± 0.02	0.76 ± 0.04	0.30 ± 0.02	0.24 ± 0.04	0.75 ± 0.02
**4b**	0.43 ± 0.01	0.41 ± 0.01	0.35 ± 0.02	0.12 ± 0.01	0.43 ± 0.01
**4c**	0.84 ± 0.09	0.97 ± 0.07	0.73 ± 0.06	0.23 ± 0.05	1.52 ± 0.13
**4d**	0.75 ± 0.03	0.90 ± 0.09	0.82 ± 0.09	0.36 ± 0.03	1.97 ± 0.16
**4e**	0.80 ± 0.03	0.28 ± 0.02	0.40 ± 0.03	0.49 ± 0.03	0.77 ± 0.02
**4f**	1.32 ± 0.10	1.32 ± 0.10	0.37 ± 0.06	0.17 ± 0.01	1.30 ± 0.05
cisplatin	1.52 ± 0.19	5.02 ± 0.23	0.81 ± 0.02	2.03 ± 0.11	3.24 ± 0.21

## Data Availability

Data Supporting obtained results can be obtained from the authors upon request.

## Supplementary Materials

The following are available online, Figure S1: [Ru(η^6^-benzene)Cl_2_{Ph_2_P(CH_2_)_3_SPh-κ*P*}] 2a, Figure S2: [Ru(η^6^-benzene)Cl{Ph_2_P(CH_2_)_3_SPh-κ*P,*κ*S*}]PF_6_ 4a*,* Figure S3: [Ru(η^6^-indane)Cl_2_{Ph_2_P(CH_2_)_3_SPh-κ*P*}] **2c**, Figure S4: [Ru(η^6^-indane)Cl{Ph_2_P(CH_2_)_3_SPh-κ*P*,κ*S*}]PF_6_ **4c**, Figure S5: [Ru(η^6^-thn)Cl_2_{Ph_2_P(CH_2_)_3_SPh-κ*P*}] **2d**, Figure S6: [Ru(η6-thn)Cl{Ph_2_P(CH_2_)_3_SPh-κ*P*,κ*S*}]PF_6_ **4d**, Figure S7: [Ru(η^6^-mesitylene)Cl{Ph_2_P(CH_2_)_3_SPh-κ*P*,κ*S*}]Cl **3b**, Figure S8: [Ru(η^6^-mesitylene)Cl{Ph_2_P(CH_2_)_3_SPh-κ*P*,κ*S*}]PF_6_ **4b**, Figure S9: [Ru(η^6^-1,4-dialin)Cl{ Ph_2_P(CH_2_)_3_SPh-κ*P,*κ*S*}]Cl **3e**, Figure S10: [Ru(η^6^-1,4-dialin)Cl{ Ph_2_P(CH_2_)_3_SPh-κ*P,*κ*S*}]PF_6_ **4e**.

## Sample Availability

Samples of the compounds reported herein are available from the authors.
