# Supplementary material for: Arene Ruthenium(II) Complexes Bearing the κ-P or κ-P,κ-S Ph2P(CH2)3SPh Ligand
Source: Molecules. 2021 Mar 25;26(7):1860. doi: 10.3390/molecules26071860 (PMC8036862; doi:10.3390/molecules26071860)
Supplement: Supplementary file 1 [file molecules-26-01860-s001.pdf]

# Arene ruthenium(II) complexes bearing the κ-*P* or κ-*P*,κ-*S* e$Ph_2P(CH_2)_3SPh$ ligand

**Sören Arlt [1], Vladana Petković [2], Gerd Ludwig [1], Thomas Eichhorn [3], Heinrich Lang [4], Tobias Rüffer [4], Sanja Mijatović [2], Danijela Maksimović-Ivanić [2] and Goran N. Kaluđerović [3,5]***

[1] Institute of Chemistry, Martin Luther University Halle-Wittenberg, Kurt-Mothes-Straße 2, D-06120 Halle, Germany; soeren.arlt@web.de, ludwig.gerd7@gmail.com

[2] Institute for Biological Research "Sinisa Stankovic" National Institute of Republic of Serbia, University of Belgrade, Bulevar despota Stefana 142, 11060 Belgrade, Serbia; vladanap@yahoo.com, sanja-mama@ibiss.bg.ac.rs, nelamax@ibiss.bg.ac.rs

[3] Department of Engineering and Natural Sciences, University of Applied Sciences Merseburg, Eberhard-Leibnitz-Strasse 2, DE-06217 Merseburg, Germany; thomas.eichhorn@hs-merseburg.de, goran.kaluderovic@hs-merseburg.de

[4] Institute of Chemistry, Chemnitz University of Technology, Straße der Nationen 62, D-09111 Chemnitz, Germany; heinrich.lang@chemie.tu-chemnitz.de, tobias.rueffer@chemie.tu-chemnitz.de

[5] Faculty of Pharmacy, Novi Sad University of business academy in Novi Sad, Trg mladenaca 5, 21000 Novi Sad, Serbia; goran.kaluderovic@faculty-pharmacy.com

* Correspondence: goran.kaluderovic@hs-merseburg.de

## Supporting Information

### 1. Analytical data

List of HSQC-NMR spectra:

Figure S1. [Ru($\eta^6$-benzene)$Cl_2${$Ph_2P(CH_2)_3SPh$-κ*P*}] **2a**.

Figure S2. [Ru($\eta^6$-benzene)Cl{$Ph_2P(CH_2)_3SPh$-κ*P*,κ*S*}]PF6 **4a**.

Figure S3. [Ru($\eta^6$-indane)$Cl_2${$Ph_2P(CH_2)_3SPh$-κ*P*}] **2c**.

Figure S4. [Ru($\eta^6$-indane)Cl{$Ph_2P(CH_2)_3SPh$-κ*P*,κ*S*}]$PF_6$ **4c**.

Figure S5. [Ru($\eta^6$-thn)$Cl_2${$Ph_2P(CH_2)_3SPh$-κ*P*}] **2d**.

Figure S6. [Ru($\eta^6$-thn)Cl{$Ph_2P(CH_2)_3SPh$-κ*P*,κ*S*}]$PF_6$ **4d**.

Figure S7. [Ru($\eta^6$-mesitylene)Cl{$Ph_2P(CH_2)_3SPh$-κ*P*,κ*S*}]Cl **3b**.

Figure S8. [Ru($\eta^6$-mesitylene)Cl{$Ph_2P(CH_2)_3SPh$-κ*P*,κ*S*}]$PF_6$ **4b**.

Figure S9. [Ru($\eta^6$-1,4-dialin)Cl{ $Ph_2P(CH_2)_3SPh$-κ*P*,κ*S*}]Cl **3e**.

Figure S10. [Ru($\eta^6$-1,4-dialin)Cl{ $Ph_2P(CH_2)_3SPh$-κ*P*,κ*S*}]$PF_6$ **4e**.

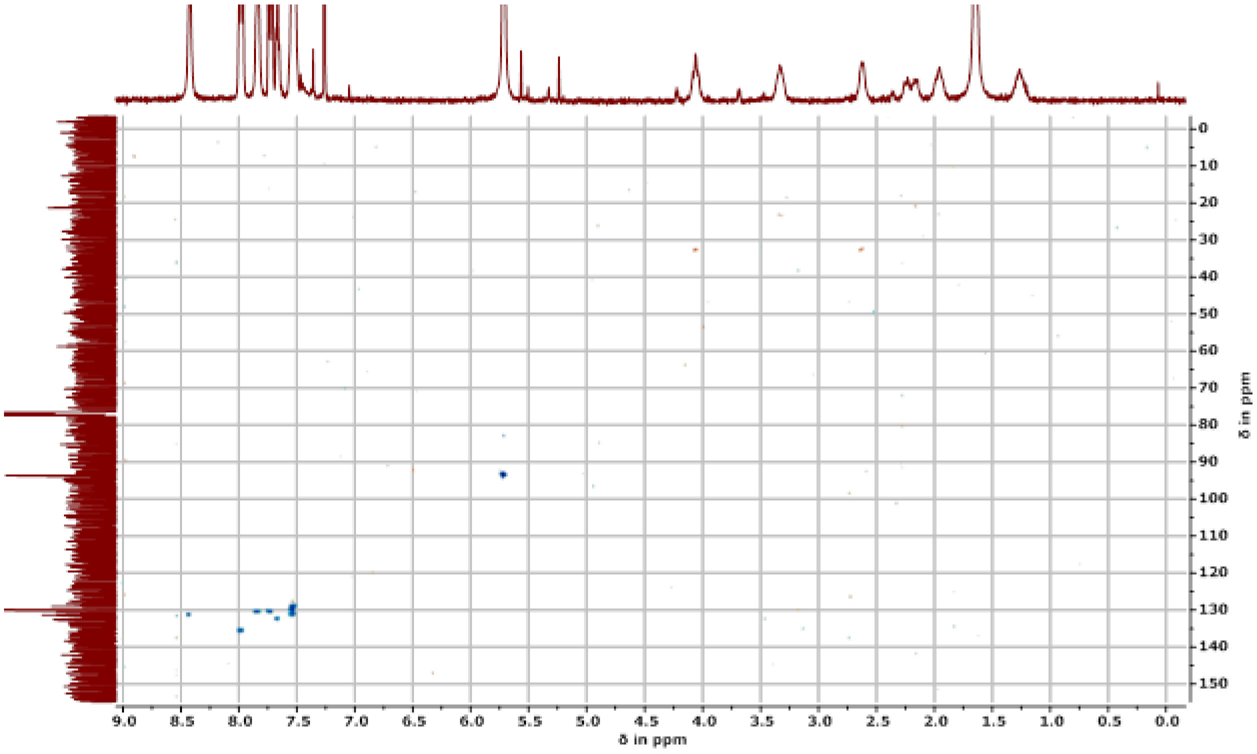


**Figure S1.** HSQC-NMR spectra for compound **2a**.

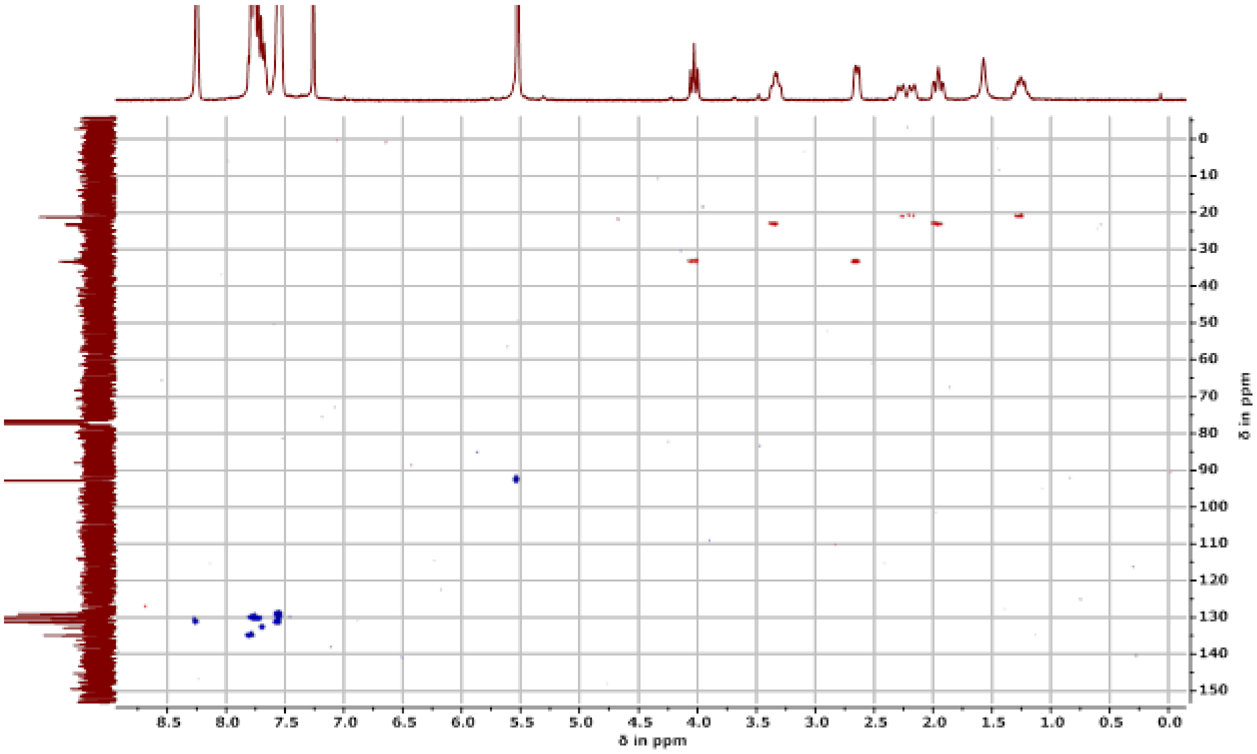


**Figure S2.** HSQC-NMR spectra for compound **4a**.

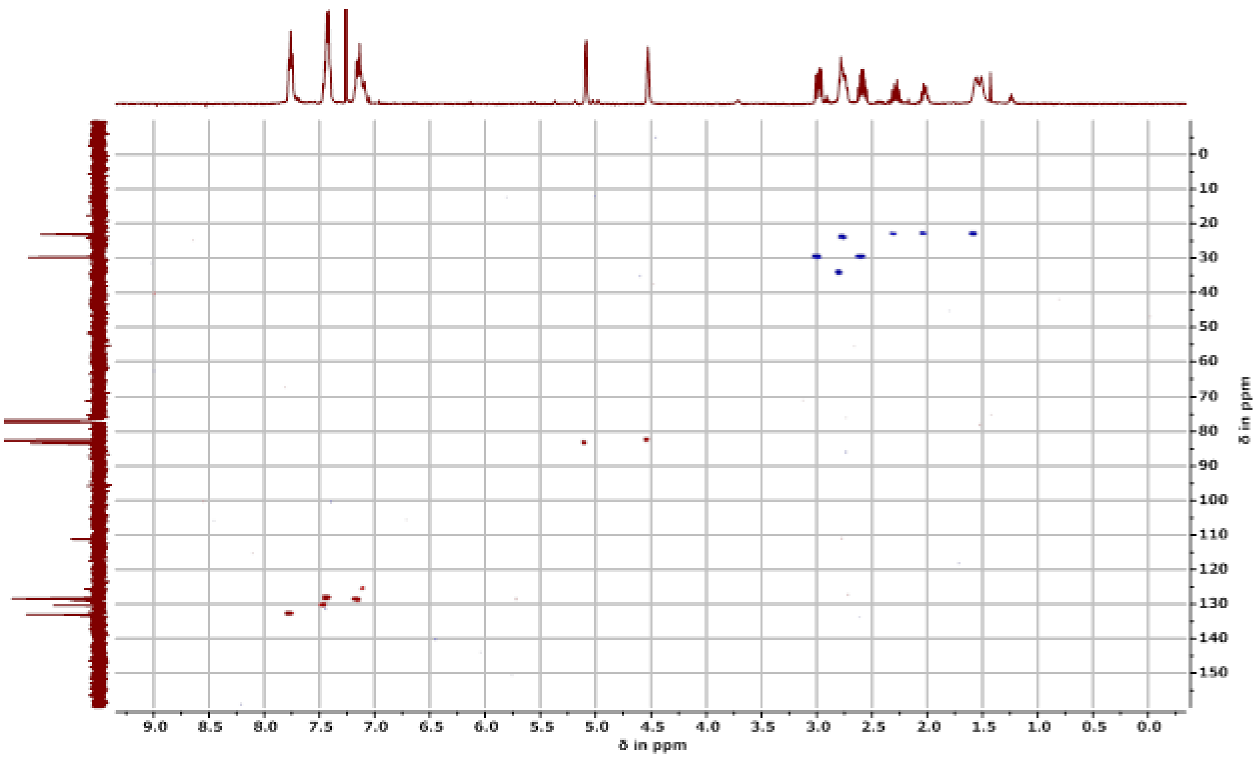


**Figure S3.** HSQC-NMR spectra for compound **2c**.

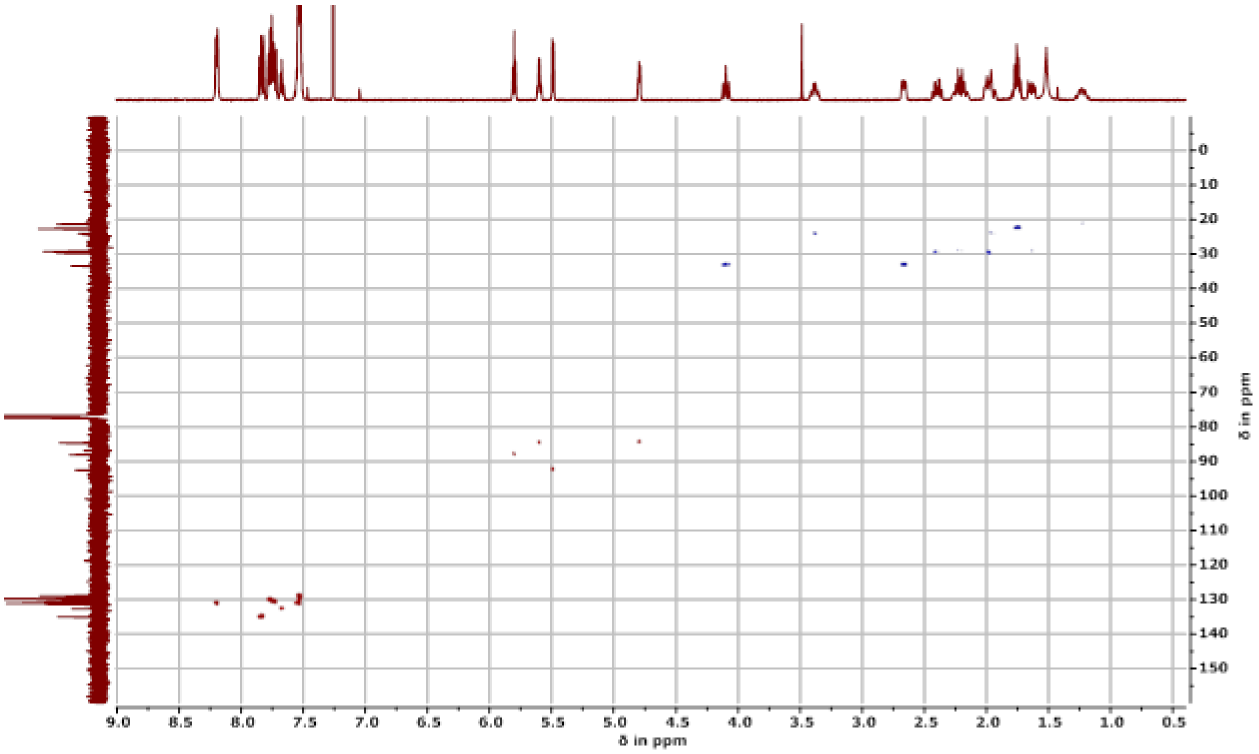


**Figure S4.** HSQC-NMR spectra for compound **4c**.

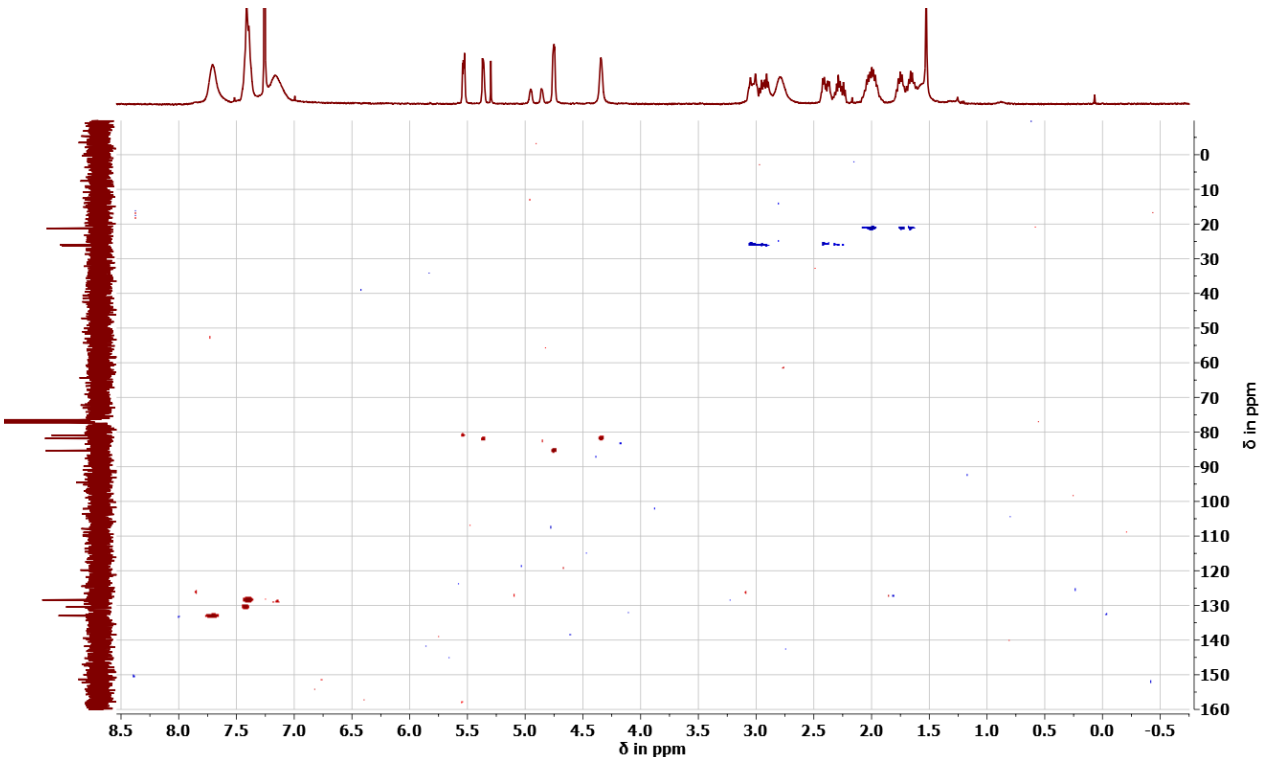


**Figure S5.** HSQC-NMR spectra for compound **2d**.

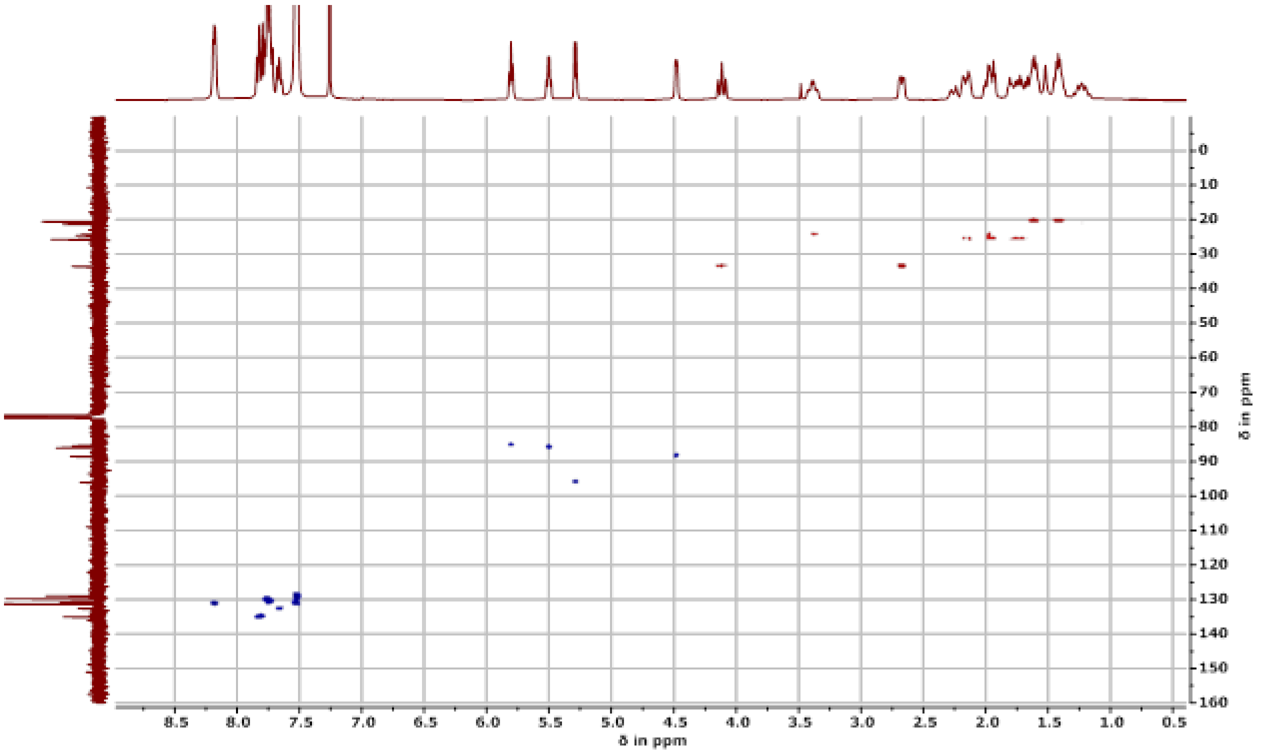


**Figure S6.** HSQC-NMR spectra for compound **4d**.

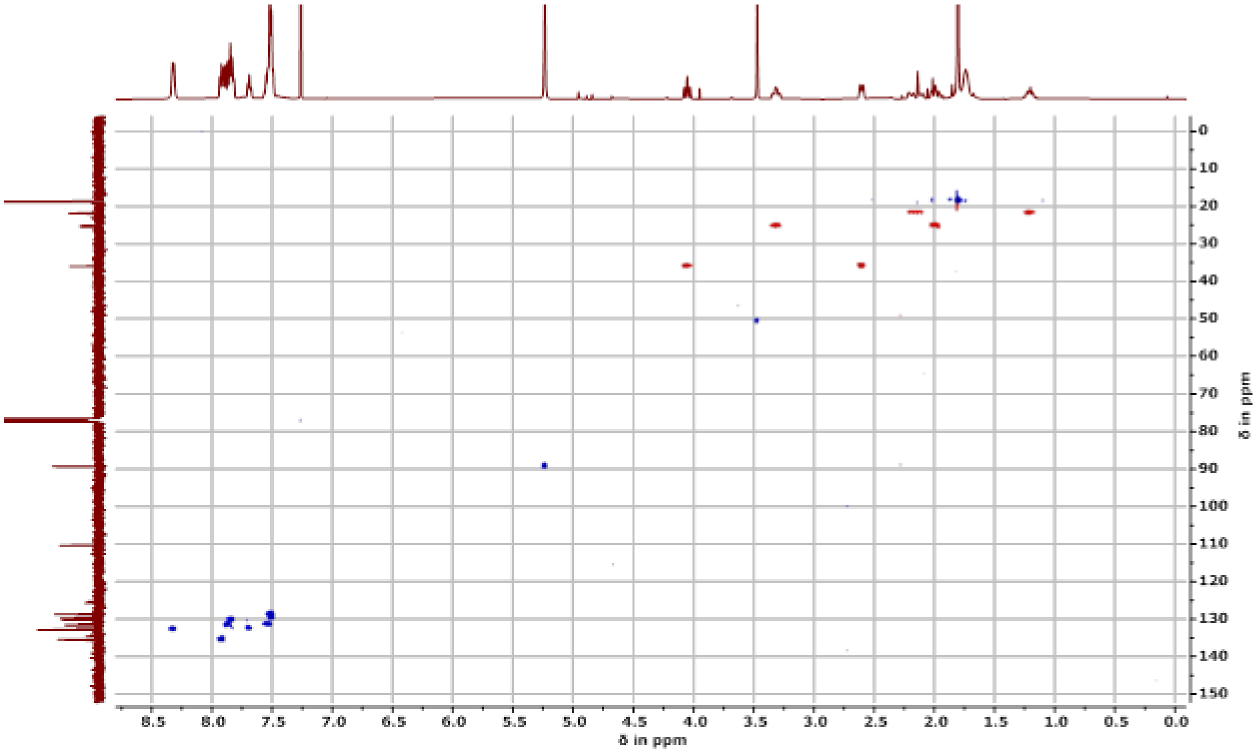


**Figure S7.** HSQC-NMR spectra for compound **3b**.

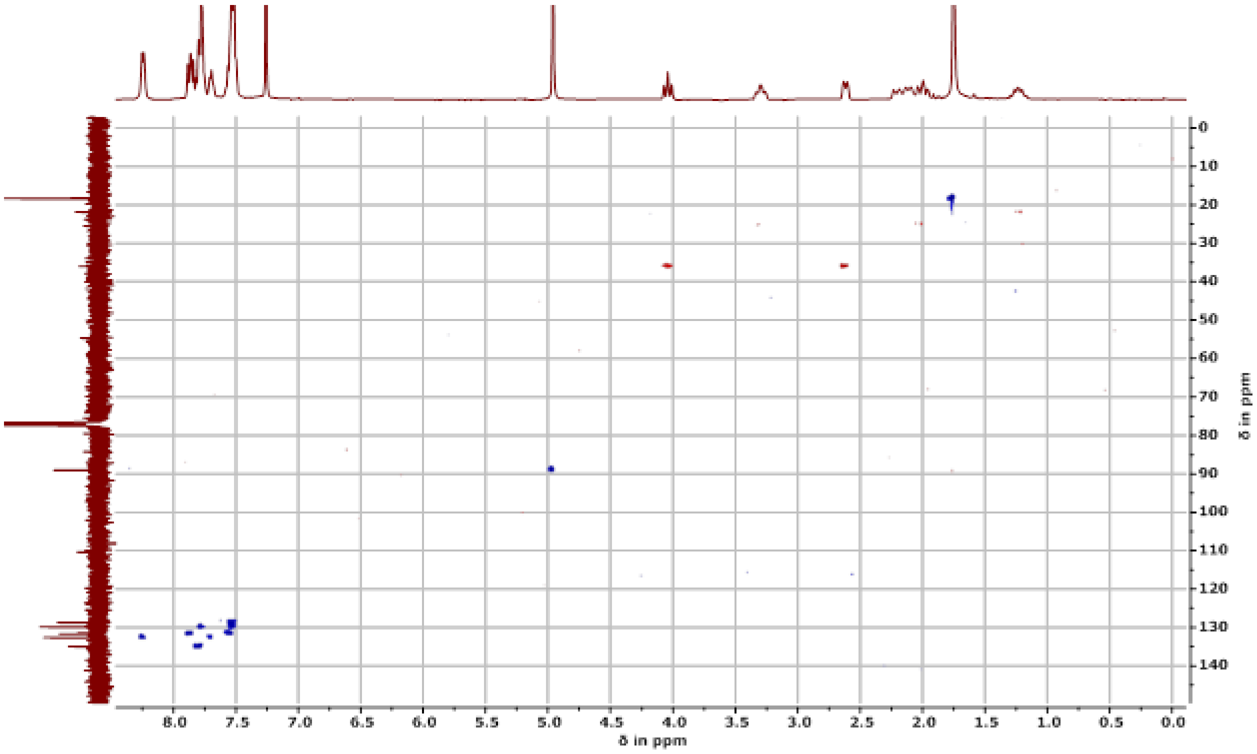


**Figure S8.** HSQC-NMR spectra for compound **4b**.

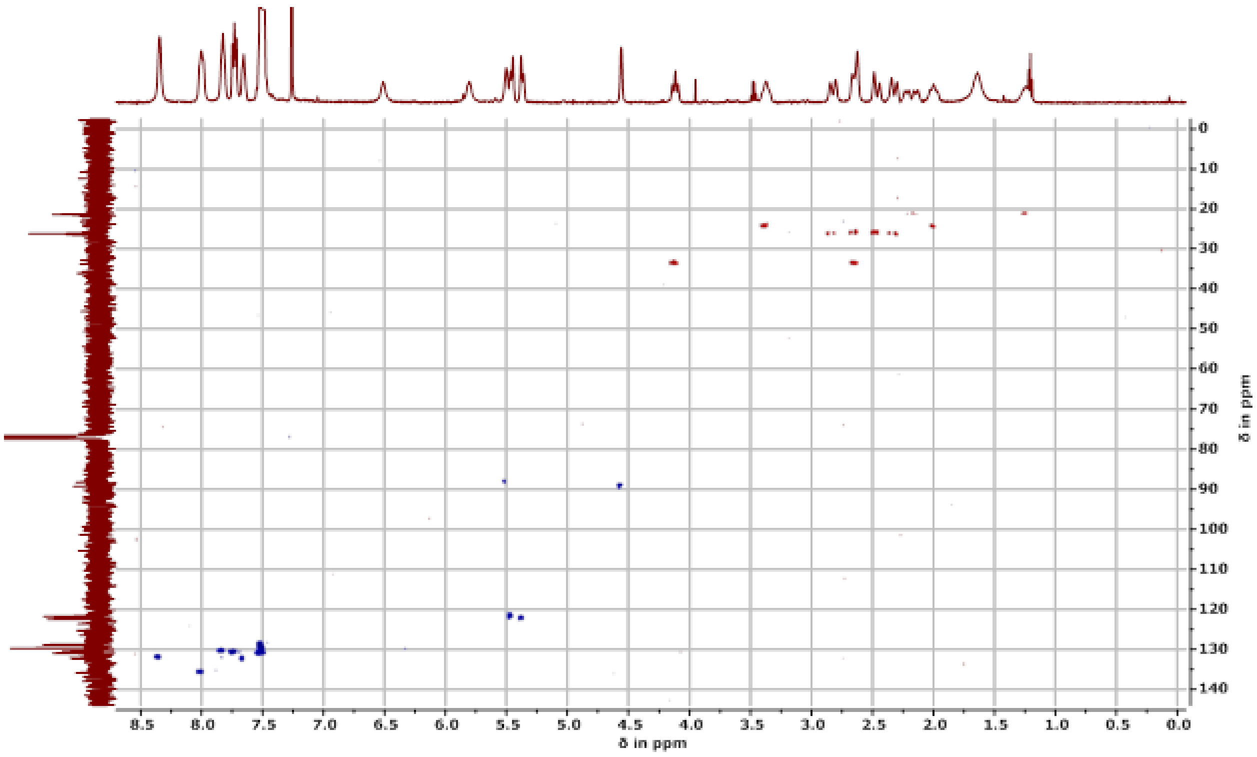


**Figure S9.** HSQC-NMR spectra for compound **3e**.

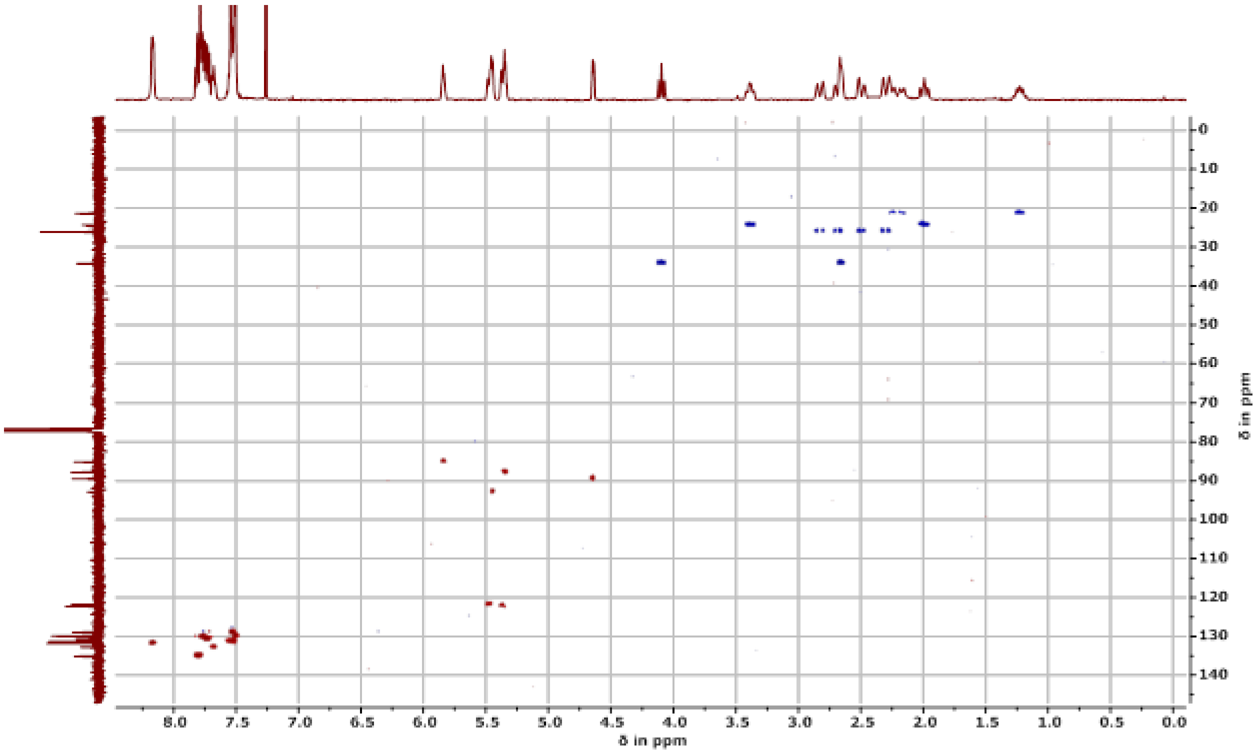


**Figure S10.** HSQC-NMR spectra for compound **4e**.
